# Quantification of 8-*oxoG* in Plant Telomeres

**DOI:** 10.3390/ijms23094990

**Published:** 2022-04-30

**Authors:** Claudia Castillo-González, Borja Barbero Barcenilla, Pierce G. Young, Emily Hall, Dorothy E. Shippen

**Affiliations:** Department of Biochemistry & Biophysics, Texas A&M University, College Station, TX 77843, USA; castillo.cm@tamu.edu (C.C.-G.); bbarbero@tamu.edu (B.B.B.); pyoung2590@gmail.com (P.G.Y.); emilyhall@tamu.edu (E.H.)

**Keywords:** *Arabidopsis*, telomeres, light stress, reactive oxygen species, 8-*oxoG*

## Abstract

Chemical modifications in DNA impact gene regulation and chromatin structure. DNA oxidation, for example, alters gene expression, DNA synthesis and cell cycle progression. Modification of telomeric DNA by oxidation is emerging as a marker of genotoxic damage and is associated with reduced genome integrity and changes in telomere length and telomerase activity. 8-oxoguanine (8-*oxoG*) is the most studied and common outcome of oxidative damage in DNA. The G-rich nature of telomeric DNA is proposed to make it a hotspot for oxidation, but because telomeres make up only a tiny fraction of the genome, it has been difficult to directly test this hypothesis by studying dynamic DNA modifications specific to this region in vivo. Here, we present a new, robust method to differentially enrich telomeric DNA in solution, coupled with downstream methods for determination of chemical modification. Specifically, we measure 8-*oxoG* in *Arabidopsis thaliana* telomeres under normal and oxidative stress conditions. We show that telomere length is unchanged in response to oxidative stress in three different wild-type accessions. Furthermore, we report that while telomeric DNA comprises only 0.02–0.07% of the total genome, telomeres contribute between 0.2 and 15% of the total 8-*oxoG*. That is, plant telomeres accumulate 8-*oxoG* at levels approximately 100-fold higher than the rest of the genome under standard growth conditions. Moreover, they are the primary targets of further damage upon oxidative stress. Interestingly, the accumulation of 8-*oxoG* in the chromosome body seems to be inversely proportional to telomere length. These findings support the hypothesis that telomeres are hotspots of 8-*oxoG* and may function as sentinels of oxidative stress in plants.

## 1. Introduction

Telomeres are dynamic nucleoprotein structures that physically cap the ends of linear chromosomes. Functional telomeres are essential for genome integrity, solving both the end-protection [1,2] and end-replication [3,4] problems. Telomeres provide end protection by sequestering chromosome ends from DNA repair machinery to prevent the activation of a DNA damage response that would otherwise cause end-to-end chromosome fusions. The end-replication problem is solved by telomerase, the reverse transcriptase dedicated to maintaining telomere repeats on chromosome termini. Telomeres typically consist of long tracts of G-rich repetitive DNA. Telomere length homeostasis is essential for viability; critically short telomeres can lead to genome instability, while abnormally long telomeres are associated with cancer proliferation [5].

As in the chromosome body, telomeres are subject to environmental cues triggering DNA modifications such as methylation and oxidation. Such modifications can profoundly alter chromatin structure and affect telomeric function [6,7,8]. Histone and DNA methylation, for example, are required for telomere maintenance in mammals [9] and genome-wide loss of DNA methylation in plants leads to telomere shortening and stem cell death [10]. Similarly, DNA can be oxidized when the levels of reactive oxygen species (ROS) are too high [11]. ROS naturally mediate cell signaling during development and in response to biotic and abiotic assaults, and are regulated via enzymatic and low molecular weight antioxidants in the cell [12]. At elevated levels, ROS can lead to oxidation of all the DNA bases, but the most studied are 8-oxoguanine (8-*oxoG*) [13] and thymine glycol (Tg) [14] adducts. Typically, 8-*oxoG* is removed by base excision repair (BER). If not repaired, 8-*oxoG* can result in GC-to-TA transversions, single-strand breaks, replication fork stalling and genome instability [6,15]. At telomeres, 8-*oxoG* inhibits telomerase activity, deregulates telomere length and impairs telomere function [6,16,17].

*Arabidopsis thaliana* is the premier model for plant telomere biology. Unlike animals and fungi, *A. thaliana* can survive the loss of essential telomeric components for multiple generations [18,19,20], offering a collection of viable mutants relevant for telomere studies. The vast collection of genetic and molecular resources for *Arabidopsis* include over a thousand fully sequenced wild-type accessions from three continents displaying significant natural variation, including in telomere length [21,22,23,24].

The study of epigenetic modifications at telomeres promises to reveal unique insight into the interplay between the environment and telomere function. However, the highly repetitive nature of telomeres and their low abundance relative to the chromosome body have precluded quantitative analysis of telomere-specific modifications in vivo. Only recently, Nanopore^®^ sequencing has allowed end-to-end chromosome sequencing [25] and analysis of some epigenetic modifications [26]. However, this method is still inaccessible for most laboratories, due to its financial cost and the extensive computational power required to process the massive amount of data generated. Most recently, the integrative analysis of *A. thaliana* through Illumina whole genome sequencing datasets enabled the assembly of the subtelomere–telomere boundaries for most chromosome ends. While this work has revealed DNA modifications (i.e., cytosine methylation) at those regions and up to 70 nt into the telomeres [27], most of the telomeric tract remains recalcitrant to chemical analysis.

Here, we sought to develop a cost-effective and reproducible method to estimate the abundance of chemical modifications at telomeres in *Arabidopsis*. Using our technique, we experimentally tested the long-standing hypothesis that telomeres are hotspots for 8-*oxoG* modification. Our method extends the gold standard for telomere length determination, terminal restriction fragment analysis (TRF), in which telomeres are differentially enriched after enzymatic digestion of genomic DNA. Telomere enrichment is coupled with a commercial ELISA-based 8-*oxoG* quantification assay to measure telomeric DNA oxidation. We show that under optimal environmental conditions, telomeres accumulate 8-*oxoG* at levels approximately 100-fold higher than the rest of the genome, and they are the primary targets of further damage upon oxidative stress. These data indicate that *Arabidopsis* telomeres are hotspots for oxidation.

## 2. Results and Discussion

### 2.1. Differential Enrichment of Telomeric DNA

**Figure 1:**
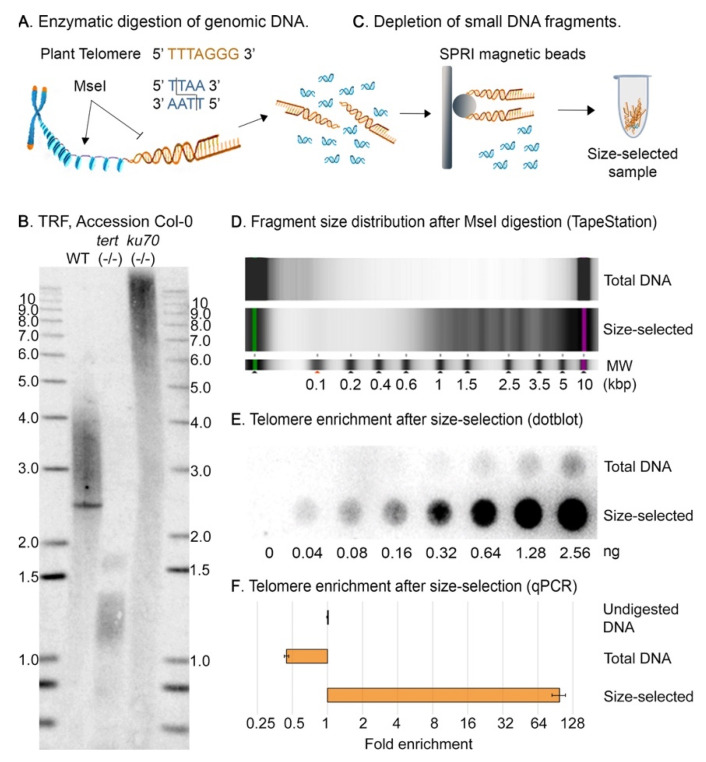
Differential telomere enrichment in solution. (**A**) Schematic representation of plant telomere-biased DNA digestion with *Mse*I. *Mse*I cannot recognize the canonical plant telomere repeat, yielding telomeric DNA fragments that are longer than the average size of the rest of the genome. (**B**) TRF showing the longer telomere length of *ku70* mutant as compared to wild-type in accession Col-0. The telomerase null mutant (*tert*) is shown for further comparison. *tert* mutants cannot extend their chromosome ends and have extremely short telomeres. Southern blot with a ^32^P-radiolabeled telomeric DNA probe. (**C**) Schematic representation of the telomere enrichment process using the SPRIselect magnetic beads (Beckman Coulter, Brea, CA, USA). Short incubation time with a low bead-to-sample ratio allows for the selective retention of long DNA molecules, depleting the sample of most of the digested non-telomeric DNA. (**D**) TapeStation outputs displaying the fragment size distribution in the initial (genomic, D5000) and final (telomere-enriched, HS D5000) samples obtained from a *ku*70 mutant. (**E**) Dot blot of a two-fold dilution series of the initial and final *ku70* samples, hybridized with a ^32^P-radiolabeled telomeric DNA probe, demonstrates telomere enrichment after depletion of small DNA fragments in the *Mse*I-digested samples. (**F**) Telomeric enrichment after size selection determined by qPCR of the telomeric repeat in *Mse*I-digested samples, and calculated with the 2^−ΔCq^ method using undigested genomic DNA as control (Cq is cycle of quantification, also known as Ct or threshold cycle).

To obtain a telomere-enriched DNA sample, we sought to extend the TRF method for telomere length determination from size separation in gel to in solution. The underlying principle is the selective digestion of non-telomeric DNA. The plant telomeric repeat, 5′-TTTAGGG-3′, is not recognized by the restriction enzyme *Mse*I, which cuts after the first nucleotide in the tetramer 5′-TTAA-3′. Digestion of genomic DNA with *Mse*I reduces the majority of genomic DNA to fragments of ca. 100 bp, while leaving telomeric DNA largely intact (Figure 1A). In the TRF protocol, *Mse*I-digested DNA is size-resolved by agarose gel electrophoresis, and telomeres are detected by Southern blot using a telomere-specific probe (Figure 1B).

We enriched telomeric DNA in solution by repurposing the commercially available solid phase reversible immobilization (SPRI) methodology, originally developed for the cleanup and homogenization of next-generation sequencing DNA libraries. We employed SPRIselect magnetic beads (Beckman Coulter, Brea, CA, USA) after *Mse*I digestion to enrich for DNA fragments of high molecular weight (Figure 1C).

Telomeres comprise an exceedingly small fraction of the total genome. Considering the *A. thaliana* Col-0 accession has telomeres ranging from 2–5 kbp (Figure 1B) [22], and the haploid genome size is ~135 Mbp, telomeres constitute a mere ~0.02% of its DNA [28]. As proof of concept, we initially looked for a telomere-enriched genome that would facilitate the visualization and tracking of large DNA molecules after *Mse*I digestion. We used the AT1G16970 *ku70* null mutant, which has ultra-long telomeres (>12 kbp) as compared with the Col-0 wild-type plants (Figure 1B) [29]. The *ku70* mutant genome contains ~0.09% telomeric repeats.

First, 50 μg of genomic DNA (measured by NanoDrop, ThermoFisher Scientific, Wilmington, DE, USA) was digested with *Mse*I (New England Biolabs, Ipswich, MA, USA) in a 200 μL reaction at 37 °C overnight. From the digestion reaction, 18 μL was set aside as the initial “total DNA” sample, and the remaining 180 μL was used for the telomere enrichment process. We followed the “left-side selection protocol” to retain fragments larger than 400 bp, as per the manufacturer instructions. We used the Agilent 2200 TapeStation with either a D5000 or a High Sensitivity D5000 ScreenTape to monitor digestion efficiency and removal of DNA fragments, respectively. In the “total DNA” sample, we observed a dominant peak at ~50–200 bp as well as a few high molecular weight minor peaks; while in the size-selected sample, the dominant fragment size retained was larger than 1000 bp (Figure 1D and Figure S2A).

Telomere enrichment was assessed using either a dot blot in a two-fold dilution series with a ^32^P-radiolabeled telomeric probe (Figure 1E) or qPCR as previously described (Figure 1F), [30]. Notably, the two-fold dilution series in the dot blot shows an approximate telomere enrichment of ≥2^6^, similar to the enrichment quantified by qPCR (Figure 1E,F).

**Figure 2:**
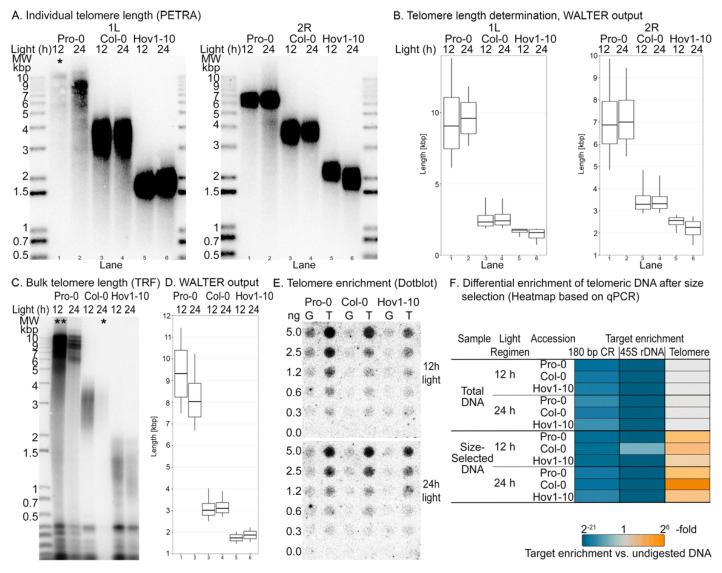
Telomeric DNA is specifically enriched by digestion and size-selection. (**A**) Assessment of telomere length of individual chromosome arms by PETRA. Top, chromosome 1, left arm (5′). Bottom, chromosome 2, right arm (3′). Telomeric DNA amplicons were detected by Southern blot with a ^32^P-radiolabeled telomere probe. (**B**) Determination of telomere length from PETRA signal in panel (**A**) using the online tool, WALTER (49). (**C**) Assessment of bulk telomere length by TRF. Telomeric DNA was detected by Southern blot with a ^32^P-radiolabeled telomere probe. (**D**) Determination of mean telomere length from TRF in panel (**C**) using the online tool, WALTER (49). (**E**) Dot blot of a two-fold dilution series of the initial and final samples, hybridized with a ^32^P-radiolabeled telomeric DNA probe demonstrates the telomere enrichment after depletion of small DNA fragments in the *Mse*I-digested samples. G denotes for total *Mse*I digested DNA (initial sample), and T denotes size-selected DNA (final sample). (**F**) qPCRs for centromeric (180 bp), ribosomal (45S rDNA), and telomeric repeats in total digested DNA and in size-selected samples. Enrichment was calculated with the 2^−ΔCq^ method, using total undigested DNA as control (Cq is cycle of quantification, also known as Ct or threshold cycle). Single asterisk (*) denotes lanes with low intensity signal, double asterisk (**) denotes an overloaded sample. We provide higher and lower intensity visuals in Figure S1 to improve the readability of results in the marked lanes.

Next, we tested the efficiency of our telomere-enrichment method in wild-type plants, taking advantage of the natural telomere length variation of *A. thaliana*. We chose three independent wild-type accessions, Hov1-10, Col-0 and Pro-0, with different telomere length setpoints (Figure 2A–D). Pro-0 telomeres are on the long end of the spectrum for *A. thaliana* and range from 5 kbp to over 12 kbp, with an average of 9.3 kbp [22], while Hov1-10 harbors some of the shortest known telomere tracts, spanning only 0.5–2.5 kbp [22]. First, we compared the telomeric DNA content before and after size selection in the three samples by dot blot using a radiolabeled telomere-specific probe in a two-fold dilution series (Figure 2E). We observed that the signal intensity was consistent with telomere length: that is, telomere content. As such, the signal in all samples from Pro-0 is greater than the signal from Col-0, which is greater than Hov1-10. Moreover, the increase in telomeric DNA after size-selection was evident in all cases, demonstrating that our protocol can be used to enrich telomeres in the 1.5 kbp to 12 kbp range.

We assessed the enrichment of other repetitive and abundant DNA after size selection using qPCR to quantify telomeric DNA, 45S rDNA and the 180 bp centromeric repeat, as previously described [30,31,32] in the total undigested genomic DNA, total *Mse*I digested and size-selected samples (Figure 2F). We successfully amplified the three targets in the undigested DNA samples but were unable to amplify the 45S rDNA and the 180 bp centromeric repeats in the *Mse*I-digested samples—a required step in our proposed protocol. This is not surprising given the high frequency of the TTAA tetramer in the *A. thaliana* genome. In some samples, we observed a lower Cq (cycle of quantification, also known as Ct or threshold cycle) value for the 45S rDNA in the size-selected versus total *Mse*I-digested samples, implying some enrichment of this sequence. Recently, Farrel et. al. demonstrated that *A. thaliana* rDNA sequences are heavily populated by interstitial telomeric repeats, many of which are degenerate [27] and can be cut by *Mse*I. However, intact telomeric sequences can be enriched by our protocol, resulting in the lower threshold of detection. Thus, while rDNA contains some intended DNA targets that can be enriched in the size-selection step, *Mse*I digestion efficiently depletes most interstitial sequences, so that there is no 45S-rDNA enrichment relative to undigested DNA. Taken together, our protocol produces samples in which telomeric DNA is highly enriched, while other abundant repeats are not.

### 2.2. Quantification of Telomeric 8-oxoG

To determine how much of the 8-*oxoG* in the DNA is contributed by telomeres, we measured DNA modifications in two samples with differential telomere enrichment from the original DNA preparation.

Given

nt≡nucleotides in the sample

$$G_{t}\equiv \mathrm{Guanine}\;\mathrm{in}\;\mathrm{telomeres},\;\mathrm{and}$$

$$G_{c}\equiv \mathrm{Guanine}\;\mathrm{in}\;\mathrm{the}\;\mathrm{chromosome}\;\mathrm{body}$$

and,

$$z=\frac{8\text{-}oxoG}{nt},\;x=\frac{8\text{-}oxoG_{c}}{G_{c}},\;y=\frac{8\text{-}oxoG_{t}}{G_{t}}\;$$

For each sample (i.e., total *Mse*I-digested DNA—initial (*i*)—and telomere-enriched—final (*f*)), we established the following premises:

(1) $$8\text{-}oxoG=8\text{-}oxoG_{t}+8\text{-}oxoG_{c}$$

(2) $$z=\left(f_{c}\cdot f_{G_{c}}\cdot x\right)+\left(f_{t}\cdot f_{G_{t}}\cdot y\right)$$

(3) $$1=f_{c}+f_{t}$$

where,

$$f_{c}\equiv \mathrm{fraction}\;\mathrm{of}\;\mathrm{nucleotides}\;\mathrm{from}\;\mathrm{the}\;\mathrm{chromosome}\;\mathrm{bodies},$$

$$f_{t}\equiv \mathrm{fraction}\;\mathrm{of}\;\mathrm{nucleotides}\;\mathrm{from}\;\mathrm{telomeres},$$

$$f_{G_{c}}\equiv \mathrm{Guanine}\;\mathrm{content}\;\mathrm{in}\;\mathrm{the}\;\mathrm{chromosome}\;\mathrm{body},\;\mathrm{and}$$

$$f_{G_{t}}\equiv \mathrm{Guanine}\;\mathrm{content}\;\mathrm{in}\;\mathrm{telomeres}\cdot$$

Through our method for telomere enrichment in solution, we produce two samples from the same organism with varying telomere representation ($f_{t}$). Notably, the genomic and telomeric guanine content are constant and known, while *x* and *y* are constant but unknown. Since we can measure 8-*oxoG* in the two DNA samples, we can experimentally determine *z* for the samples. Then, with a simple algebraic manipulation it is possible to calculate *x* and *y* as follows:

For the initial sample, total *Mse*I-digested DNA:

(4) $$z_{i}=\left(f_{c_{i}}\cdot f_{G_{c}}\cdot x\right)+\left(f_{t_{i}}\cdot f_{G_{t}}\cdot y\right)$$

*z_i_*, $f_{c_{i}}\;\mathrm{and}\;f_{t_{i}}$ are known from the *A. thaliana* genome sequence and the known telomere length of the sample.

For the final sample, telomere-enriched DNA:

(5) $$z_{f}=\left(f_{c_{f}}\cdot f_{G_{c}}\cdot x\right)+\left(f_{t_{f}}\cdot f_{G_{t}}\cdot y\right)$$

*z_f_* $f_{c_{f}}\;\mathrm{and}\;f_{t_{f}}$ can be estimated by measuring telomere enrichment by qPCR, Southern blot or using the size distribution of DNA fragments as detected by TapeStation or Bioanalyzer. For the latter case, we suggest using a cutoff of 400 bp as that has been established to be the minimum length of a functional telomere in *A. thaliana* [19,22]. It is essential that telomere enrichment be calculated by measuring the telomeric fraction in the initial and final samples using the same method (Figure 1E,F, Figure S2D and Figure 2E,F).

Then, solving *z_i_* and *z_f_* for *x*, we obtain

(6) $$x=\frac{Z_{f}-\left(f_{t_{f}}\cdot f_{G_{t}}\cdot y\right)\;}{\left(f_{c_{f}}\cdot f_{G_{c}}\right)}\;\mathrm{and}\;x=\frac{Z_{i}-\left(f_{t_{i}}\cdot f_{G_{t}}\cdot y\right)}{\left(f_{c_{i}}\cdot f_{G_{c}}\right)}$$

Let us call

(7) $$Enrichment,\;\;\;\;E_{c}=\frac{f_{c_{f}}}{f_{c_{1}}},\;\mathrm{then}$$

(8) $$y=\frac{z_{f}-E_{c}\cdot z_{i}}{f_{t_{f}}\cdot f_{G_{t}}-E_{c}\cdot f_{t_{i}}\cdot f_{G_{t}}}$$

**Figure 3:**
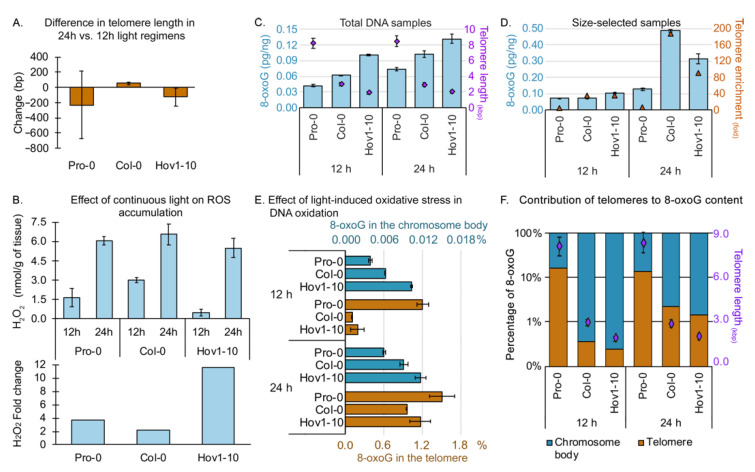
Telomeres are enriched in 8-*oxoG* and may protect the genome from oxidative damage. (**A**) Difference in telomere length in wild-type plants exposed to a 24-h photoperiod for two weeks versus control plants grown in a 12-h photoperiod. (**B**) Effect of photoperiod length on accumulation of hydrogen peroxide (H_2_O_2_) in wild-type plants. Top, H_2_O_2_ content. Bottom, fold change in H_2_O_2_ in 24-h versus 12-h photoperiod. (**C**) Determination of total 8-*oxoG* in the initial, total genomic DNA samples as measured by the ELISA-based kit (Invitrogen™, ThermoFisher Scientific, Frederick, MD, USA). Average telomere length in each sample is shown as purple diamonds plotted on the secondary *y*-axis, shown in purple on the right side. (**D**) Determination of total 8-*oxoG* in the final, telomere-enriched samples as measured by the ELISA-based kit (Invitrogen™, ThermoFisher Scientific, Frederick, MD, USA). Average telomere enrichment in each sample is shown by ochre triangles plotted on the secondary *y*-axis, shown in ochre on the right side. Enrichment was determined by qPCR with the 2^−ΔCq^ method (Cq is cycle of quantification, also known as Ct or threshold cycle) (**E**) 8-*oxoG* was calculated for telomeres (orange, bottom *x*-axis) and chromosome body (cyan, top *x*-axis) in wild-type plants from three different *A. thaliana* accessions, exposed to either 12-h or 24-h photoperiod for two weeks. (**F**) Relative contribution of telomeres (orange) and chromosome body (cyan) to total 8-*oxoG*. Average telomere length in each sample is shown as purple diamonds plotted on the secondary *y*-axis, shown in purple on the right side.

Since *y* is the fraction of 8-*oxoG* relative to telomeric guanines, and *x* is the fraction of 8-*oxoG* relative to guanines in the chromosome body, telomere and chromosome body oxidation can be calculated by multiplying *y* and *x* by $f_{G_{t}}$ or $f_{G_{c}}$, respectively (Figure 3E). These values lead to total 8-*oxoG*.

(9) $$8\text{-}oxoG_{t}=y\cdot f_{G_{t}}\cdot \left(telomere\;length\times 10\right),\;\mathrm{and}\;8\text{-}oxoG_{c}=x\cdot f_{G_{c}}\cdot Genome\;size$$

While sensitivity of the ELISA 8-*oxoG* quantification is not suitable for single base resolution, these data can be used to estimate the relative contribution of telomeres and chromosome body to genome oxidation (Figure 3F). 

Here, we quantified the amount of 8-*oxoG* present at chromosome ends, using the commercially available DNA damage competitive ELISA kit (Invitrogen™, ThermoFisher Scientific, Frederick, MD, USA). We highlight that this method of telomere enrichment can also allow the estimation of other chemical modifications at telomeres.

### 2.3. Telomeres Are Hotspots of 8-oxoG and Serve as Protectors of the Genome under Oxidative Stress

Oxidative stress consists of an increase in ROS levels that can trigger indiscriminate oxidation of proteins, lipids and nucleic acids [33]. Prolonged exposure to oxidative stress can lead to permanent DNA damage as accumulation of oxidized bases, such as 8-*oxoG*, can overwhelm the DNA repair machinery. 8-*oxoG* is the most abundant oxidation lesion and its accumulation can result in altered gene expression, inhibition of DNA synthesis and cell-cycle progression, and ultimately cell death. G-rich regions in the DNA are non-aleatorily distributed [34,35,36], and some are prone to the formation of non-canonical DNA structures (i.e., G-quadruplexes) in regulatory elements [37]. 8-*oxoG* can stabilize such conformations [38], and is proposed to function as a modulator and not simply a byproduct of oxidative stress [7]. Notably, telomeres are prone to G-quadruplex formation [39,40]. Thus, 8-*oxoG* may be an important regulatory molecule for telomere function.

Our new assay and model to estimate telomeric 8-*oxoG* provide an opportunity to test the long-standing hypothesis that telomeres are hotspots for DNA oxidation [41,42]. Telomeres have a higher GC content than the rest of the genome. For example, the GC content of mammalian telomeres is 50% GC versus ~41% in the genome [43], while plant telomeres stand at ~42% CG versus ~36% genome-wide [28]. In addition, telomeres are highly dynamic structures that engage a multitude of protein complexes that potentially make them more susceptible to chemical modification and less accessible to DNA repair [42]. If it is true that telomeres are hotspots of DNA oxidation, we expect that (i) longer telomeres contribute more 8-*oxoG* to the genome, and (ii) telomeres gain more 8-*oxoG* than the genome upon oxidative stress. To examine the relationship between telomere length and 8-*oxoG* content, we used the wild-type accessions Hov1-10, Col-0 and Pro-0 (Figure 2). Light stress is a known inducer of ROS [44], and, therefore, we exposed the three accessions to two different light regimens for a period of two weeks: a control 12:12 h light:dark and a stress-inducing 24 h of constant light (Figure 3A,B). If telomeres are hotspots of DNA oxidation, we predict that the longer telomeres in the Pro-0 accession will contribute more 8-*oxoG* to the genome than the shorter telomeres in Hov1-10, and that the 8-*oxoG* content upon oxidative stress would increase more at telomeres than in the chromosome body. 

We confirmed the induction of ROS upon continuous light by measuring the concentration of hydrogen peroxide (H_2_O_2_) in plant tissues (Figure 3B, top panel). The three accessions accumulated H_2_O_2_ at varying levels under our control light regimen (12 h); however, upon constant light, H_2_O_2_ concentration was elevated in each case, indicating that our treatment had successfully overwhelmed the ROS scavenging systems and induced oxidative stress. The differences in the basal H_2_O_2_ concentration may result in varying levels of stress induction, so we considered the H_2_O_2_ fold change upon stress for subsequent analyses (Figure 3B, bottom panel). We measured telomere length on individual chromosome arms (Figure 2A,B) and observed only minimal changes in telomere length upon light stress in the three accessions. Analysis of bulk telomere length by standard TRF (Figure 2C,D) confirmed the variant telomere-length setpoints among these accessions, and the absence of length changes upon light stress (Figure 2A–D and Figure 3A). We used the average telomere lengths calculated with these experiments to estimate $f_{t_{i}}$, considering a haploid genome of 135 Mbp and ten telomeres per haploid genome, this is $f_{t_{i}}=\frac{telomere\;length\times 10}{135\;\mathrm{Mbp}}$.

8-*oxoG* content was assessed in both total DNA and telomere-enriched samples using the DNA damage competitive ELISA kit (Invitrogen™, ThermoFisher Scientific, Frederick, MD, USA) (Figure S2), with at least two biological repeats per condition, per accession. In the three accessions, we found an increment in 8-*oxoG* in total DNA from plants exposed to 24-h versus 12-h light regimens (Figure 3C). Interestingly, the accumulation of 8-*oxoG* followed a trend inversely proportional to telomere length regardless of the light regimen. That is, the longer the telomere, the lower the accumulation of 8-*oxoG* (Figure 3C). We observed an increase in 8-*oxoG* (3–5-fold) in the size-selected (telomere-enriched) versus total DNA samples (Figure 2D). Notably, the efficiency of the telomere enrichment varied from sample to sample and the 8-*oxoG* content in the size-selected sample changed according to telomere enrichment (Figure 2F and Figure S2), suggesting telomeres accumulate more 8-*oxoG* than the chromosome body.

Under the control 12-h light regimen, we determined the chromosome body 8-*oxoG* content of Pro-0, Col-0 and Hov1-10 to be ~0.004%, 0.006% and 0.01%, respectively. Upon stress, the 24 h of light, the 8-*oxoG* content of the chromosome body in the corresponding accessions went up to ~0.006%, 0.009% and 0.012%. Considering their respective average telomere lengths as ~8.3 kbp, 3.0 kbp and 1.9 kbp, our data suggest that shorter telomeres correlate with higher genomic 8-*oxoG* regardless of the light regimen. Following the same sample order, telomeric 8-*oxoG* under control conditions was ~1.2%, 0.1% and 0.2%, and changed to ~1.51%, 0.96% and 1.17% upon light-induced oxidative stress (Figure 2E). The increments in telomere oxidation are consistent with H_2_O_2_ fold change upon 24-h light treatment (Figure 3B, bottom panel), hinting at an important contribution of telomeres to total 8-*oxoG* upon oxidative stress. 

Finally, we calculated the total 8-*oxoG* under the two light regimens to determine the relative contribution of telomeres (Figure 3F). As predicted, the telomeres of Pro-0 contributed more to the total 8-*oxoG* than those of Col-0, which contributed more than the telomeres of Hov1-10, and this trend was exacerbated upon oxidative stress. This means that telomeres gained more 8-*oxoG* than the chromosome body upon stress. 

Thus, that *Arabidopsis* telomeres appear to be hotspots for 8-*oxoG* accumulation in response to oxidative stress.

## 3. Materials and Methods

### 3.1. Plant Materials

Seeds for the natural accessions were obtained from The *Arabidopsis* Biological Resource Center (ABRC) at Ohio State University, stocks CS22649 (Pro-0), CS28994 (Col-0) and CS76931 (Hov1-10). Seeds were surface-sterilized in 2.7% sodium hypochlorite with 0.1% Triton X-100 for 10 min, stratified in 4 °C for three days, and plated on 0.8% agar plates with half-strength Murashige & Skoog minimum medium, supplemented with 1% sucrose. Seeds were germinated under a 12-h photoperiod of an average illuminance of 5000 (+/− 250) lux attained with 3000:6500 Kelvin lights in a 1:1 ratio, and at a constant temperature of 22 °C. Then, 7-day-old seedlings were transferred to soil (Sunshine, Mix 5, Sun Gro Horticulture, Agawam, MA, USA) and allowed to grow under the same conditions for three weeks. At this point, half of the plants were moved to a 24-h photoperiod chamber with an average illuminance of 8000 (+/− 400) lux, maintaining the warm-to-cool light ratio. Two weeks later, whole plants from both light regimens were harvested for subsequent analyses.

### 3.2. Quantification of Hydrogen Peroxide

To estimate ROS accumulation, we measured H_2_O_2_ as previously described [45]. Briefly, 0.3 g of plant material were homogenized in 2 mL of 0.1% (*w*/*v*) trichloroacetic acid (TCA). The mixture was clarified by centrifugation at 10,000× *g* for 20 min in 4 °C. The supernatant (ca. 0.5 mL) was neutralized by adding 0.5 mL of 0.1 M Tris-HCl (pH 7.6), and then mixed with 1 mL of 1 M potassium iodide (KI) for 1 h. H_2_O_2_ and KI react, producing H_2_O and I_2_. I_2_ can be spectrophotometrically determined by measuring absorbance at 285 nm or 350 nm. Since plant extracts produce a high absorbance background in that range, 390 nm is the standard. The H_2_O_2_ concentration was calculated using a linear regression from a standard curve ranging from 100 to 1000 μmol H_2_O_2_ mL^−1^.

### 3.3. DNA Extraction

Genomic DNA was prepared following the standard cetyltrimethylammonium bromide (CTAB) method [46] with minor modification. A total of 0.5–1 g of frozen plant DNA tissue was ground to a powder and mixed in a 1:1 ratio (*w*/*v*) with the CTAB lysis buffer (100 mM Tris-HCl pH 8.0, 20 mM EDTA pH 8.0, 1.4 M NaCl, 2% CTAB, 2% ß-mercaptoethanol) and incubated at 65 °C for 1 h; then, by adding phenol:chlorophorm:isoamyl alcohol (25:24:1, *v*/*v*/*v*) to the plant extract in a 1:1 ratio, total DNA was extracted into the aqueous phase by careful mixing. The phases were separated by centrifugation at 7500× *g* for 20 min. The aqueous phase was transferred to a new tube and DNA was precipitated for 2 h at −20 °C, by adding 3M sodium acetate (1/10 aqueous phase volume) and 100% 2-propanol (2× aqueous phase volume). DNA was pelleted by centrifugation at 17,000× *g* for 20 min, washed with 70% ethanol and treated with 10 mg/mL RNase A for 30 min at 37 °C.

### 3.4. Telomere Length Assessment

Bulk telomere length was measured by TRF analysis performed with MseI (New England Biolabs, Ipswich, MA, USA) restriction enzyme and with ^32^P 5′end-radiolabeled (T_3_AG_3_)_4_ oligonucleotide as a probe [47]. Single telomere analysis was performed using primer extension telomere repeat amplification (PETRA) as previously described [48]. Telomere length on Southern blots was measures using the online tool, WALTER [49].

### 3.5. qPCR for Quantification of Telomeric DNA, 45S rDNA and 180 bp Centromeric Repeats

Quantification of telomeric, ribosomal and centromeric DNA was performed by quantitative PCR as previously published [30,31,32], modifying the monochrome multiplex standardization to normalization by input DNA in the reaction. This modification was necessary due to our *Mse*I digestion step, which effectively removes the single-copy gene employed as an internal standard for quantification. The primer pairs were: (1) telomeric DNA, TelA (5′-CCC CGG TTT TGG GTT TTG GGT TTT GGG TTT TGG GT-3′) and TelB (5′-GGG GCC CTA ATC CCT AAT CCC TAA TCC CTA ATC CCT-3′) [30]; (2) rDNA, 18S(XbaI)F (5′-CTAGAGCTAATACGTGCAACAAAC-3′) and 18S(Hpa)R (5′-TTGCAATGATCTATCCCCATC-‘3′) [31]; and (3) 180 bp centromeric repeat, 180 F (5′-CCG TAT GAG TCT TTG GCT TTG-3′) and 180 R (5′-TTG GTT AGT GTT TTG GAG TCG-3′) [32].

The 20 μL qPCR reactions were prepared as follows: 10 μL PowerTrack SYBR™ Green Master Mix (Applied Biosystems, ThermoFisher Scientific Baltics UAB, Vilnius, Lithuania), 0.3 μL of each 10 μM Primer, 0.5 μL Yellow sample buffer (optional), 2 μL DNA (1 ng/μL) as measured by Qubit™ 4 fluorometer (ThermoFisher Scientific, Life Technologies Holdings PTE Ltd., Melaka, Malasya), and 6.9 μL DNase-free water.

The thermocycler protocols were as follows: (1) for telomeric DNA (start at 95 °C for 120 s, then twice 95 °C for 15 s and 49 °C for 15 s; then, cycle 40 times 95 °C for 15 s, 62 °C for 10 s, 74 °C for 20 s with signal acquisition; finally 95 °C for 30 s followed by 50 °C for 30 s; using a heated lid at 105 °C); (2) and (3) for ribosomal and centromeric DNA (start at 95 °C for 120 s, then twice 95 °C for 15 s followed by 49 °C for 15 s; then, cycle 40 times 95 °C for 20 s, 57°C for 30 s, 72 °C for 90 s with signal acquisition; using a heated lid at 105 °C). A melting curve from 57 °C to 95 °C at 0.5 °C/1 s was generated for each reaction product to test for amplification products.

### 3.6. Telomere Enrichment

A total of 50 μg of total genomic DNA were digested overnight with 30 U of *Mse*I in 200 μL at 37 °C. To enrich large DNA fragments after digestion, we used solid phase reversible immobilization (SPRI) methodology, SPRIselect magnetic beads (Beckman Coulter, Brea, CA, USA). According to the manufacturer’s instructions, we employed the “left-side selection protocol” in a bead-to-sample ratio of 0.4. Importantly, incubation time was kept at 1 min, since longer times resulted in higher accumulation of small DNA fragments.

### 3.7. 8-oxo-G Quantification

8-*oxoG* was measured using the DNA Damage Competitive ELISA kit (Cat. # EIADNAD, Invitrogen™, ThermoFisher Scientific, Frederick, MD, USA), following the manufacturer’s instructions.

## 4. Conclusions

Due to their low abundance, telomeres present a challenge for quantitative analyses of chemical DNA modifications. Here, we describe the development of a new technique for determination of DNA oxidation at telomeres that couples differential telomere enrichment to downstream quantitative 8-*oxoG* ELISA. We used this approach to determine the abundance of 8-*oxoG* modifications at *Arabidopsis* telomeres in three different wild-type accessions exhibiting varying telomere length under normal and oxidative stress conditions. We note that our protocol can be readily adapted to the assessment of other DNA modifications, besides oxidation.

Telomere oxidation is emerging as a marker of oxidative stress [50,51,52], and chromosome ends are hypothesized to act as the preferred target of oxidative damage modifications. Numerous in vitro studies suggest that telomeres are particularly reactive to ROS species and that telomeric guanines are more prone to oxidation compared to non-telomeric DNA with similar nucleotide content [16,17]. Repair of 8-*oxoG* at telomeres, and specifically the 3′ overhang, has been shown to be less effective as in the rest of the genome [53]. With the method described in this paper, we now provide a strategy to test in vivo the long-standing hypothesis that telomeres are hotspots for DNA oxidation in eukaryotes.

Our data reveal that plants preferentially accumulate 8-*oxoG* at telomeres, and that the rest of the genome gains 8-*oxoG* upon stress in a magnitude that seems inversely proportional to telomere length, raising the intriguing possibility that telomeres function as sentinels of oxidative stress and protectors of the genome in vivo. Given the reduced sample size, a three-point analysis, such as the one presented here, is not sufficient to draw a definitive conclusion. Notwithstanding, these data are provocative and together with our new methodology may fuel a more in-depth look at DNA oxidation dynamics from the perspective of telomere function and telomere-length homeostasis. In conclusion, our new method provides a powerful semi-quantitative, reproducible and affordable tool to allow routine assessment of telomeric DNA modifications under multiple genetic and environmental conditions.

## Supplementary Materials

The following supporting information can be downloaded at: https://www.mdpi.com/article/10.3390/ijms23094990/s1.
