# Supplementary figures and images for: Quantification of 8-oxoG in Plant Telomeres
Source: Int J Mol Sci. 2022 Apr 30;23(9):4990. doi: 10.3390/ijms23094990 (PMC9102096; doi:10.3390/ijms23094990)

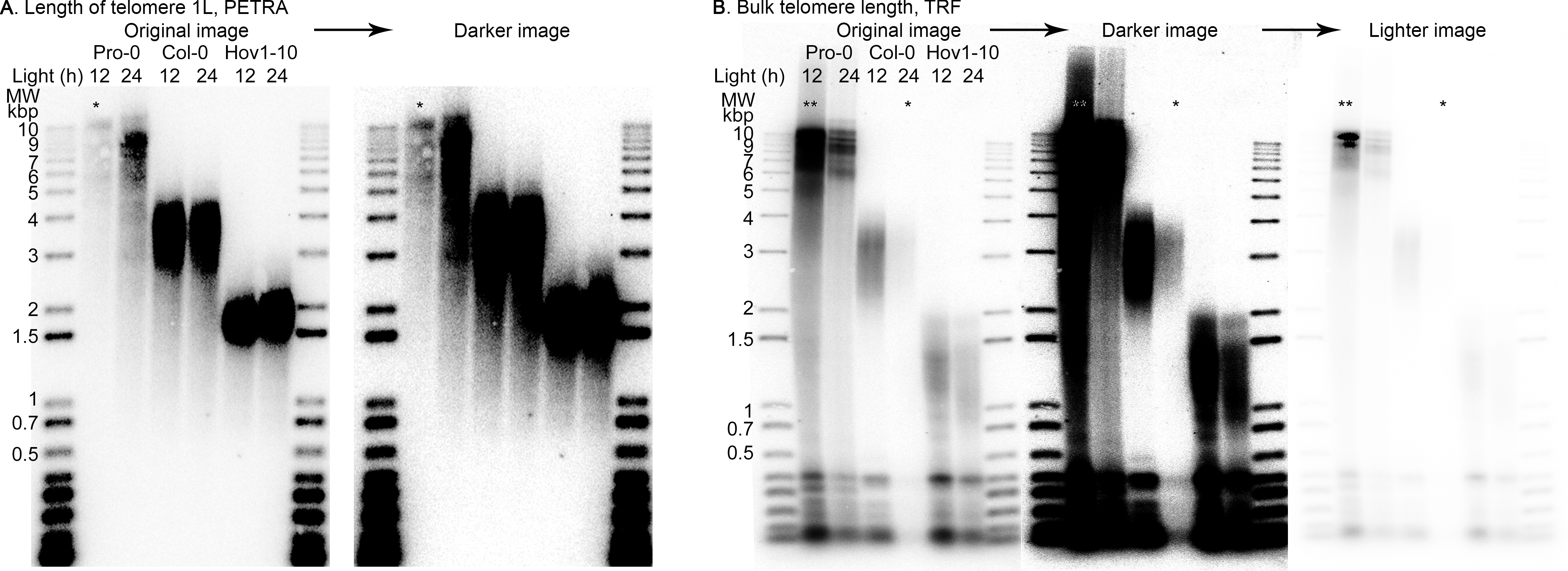

Supplement: Supplementary file 1 [file ijms-23-04990-s001.zip › Supplementary Figure S1.tif]

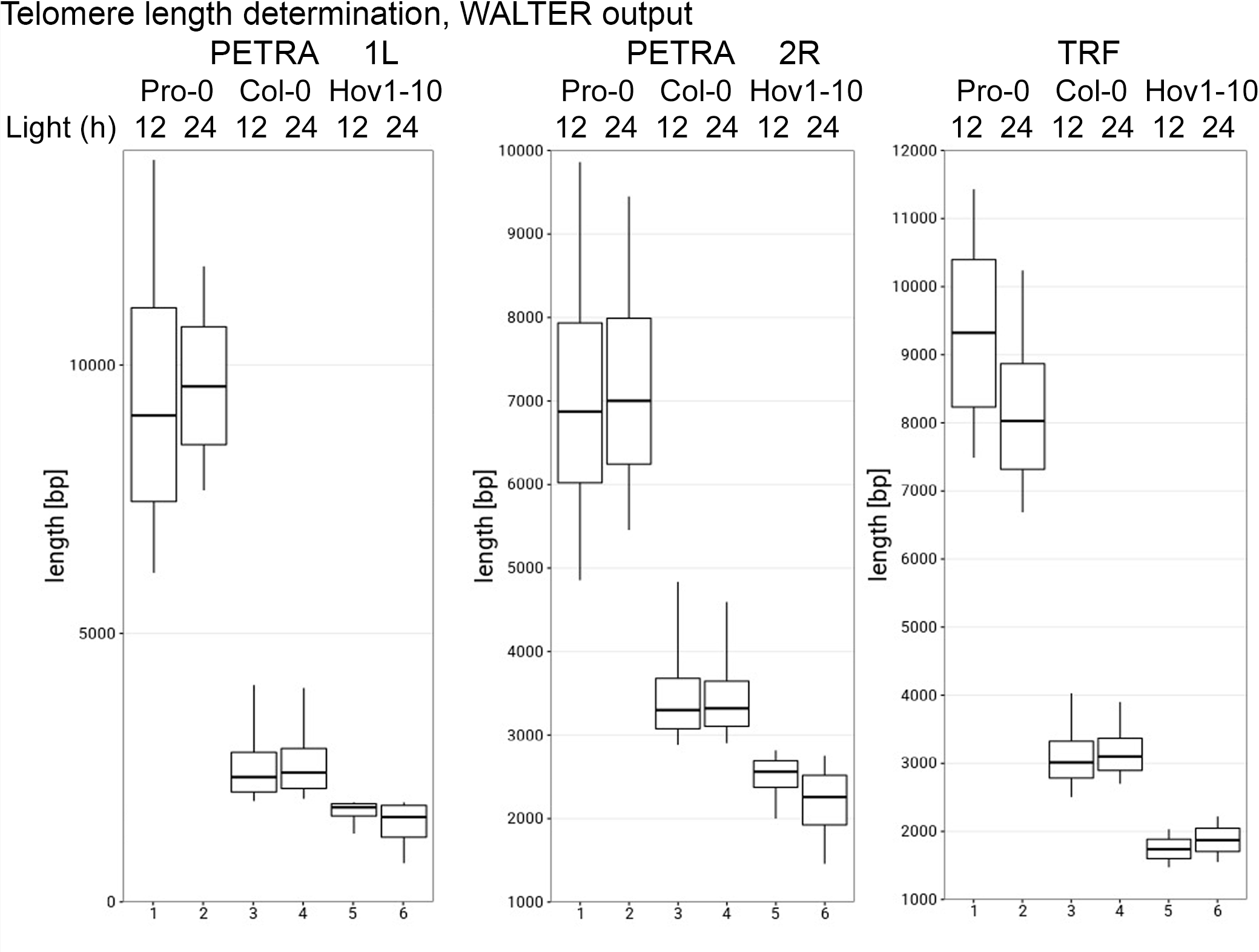

Supplement: Supplementary file 1 [file ijms-23-04990-s001.zip › Supplementary Figure S2.tif]

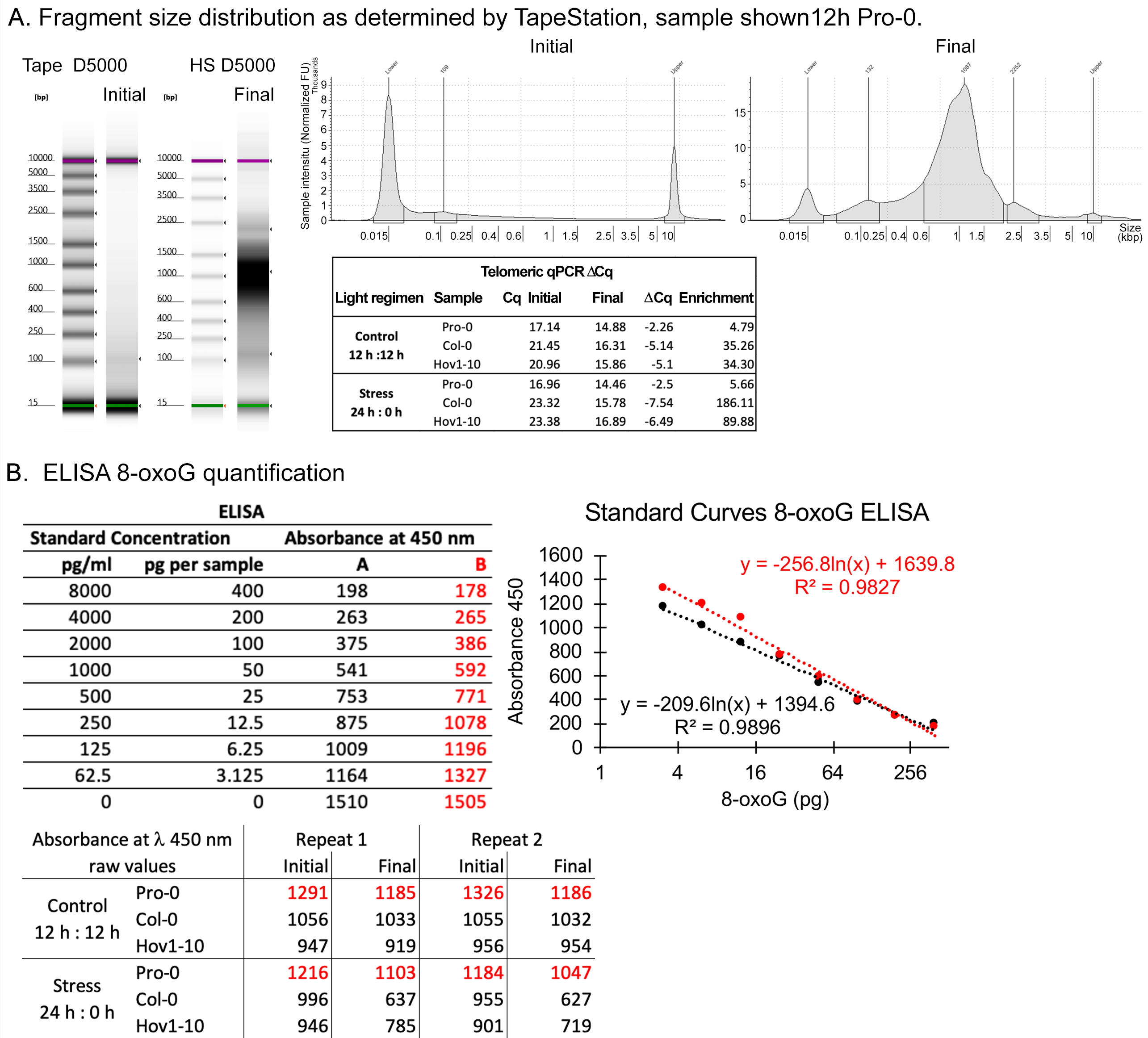

Supplement: Supplementary file 1 [file ijms-23-04990-s001.zip › Supplementary Figure S3.tif]
